# Butyrate inhibits visceral allodynia and colonic hyperpermeability in rat models of irritable bowel syndrome

**DOI:** 10.1038/s41598-019-56132-4

**Published:** 2019-12-20

**Authors:** Tsukasa Nozu, Saori Miyagishi, Rintaro Nozu, Kaoru Takakusaki, Toshikatsu Okumura

**Affiliations:** 1 Department of Regional Medicine and Education, Asahikawa Medical University, 2-1-1-1 Midorigaoka-Higashi, Asahikawa, Hokkaido, 078-8510 Japan; 2 Division of Gastroenterology and Hematology/Oncology, Department of Medicine, Asahikawa Medical University, 2-1-1-1 Midorigaoka-Higashi, Asahikawa, Hokkaido, 078-8510 Japan; 3 Research Center for Brain Function and Medical Engineering, Asahikawa Medical University, 2-1-1-1 Midorigaoka-Higashi, Asahikawa, Hokkaido, 078-8510 Japan; 4 Department of General Medicine, Asahikawa Medical University, 2-1-1-1 Midorigaoka-Higashi, Asahikawa, Hokkaido, 078-8510 Japan

**Keywords:** Pharmacology, Stress and resilience, Physiology, Irritable bowel syndrome

## Abstract

Lipopolysaccharide (LPS) or repeated water avoidance stress (WAS) induces visceral allodynia and gut hyperpermeability *via* corticotropin-releasing factor (CRF) and proinflammatory cytokines, which is a rat irritable bowel syndrome (IBS) model. As butyrate is known to suppress the release of proinflammatory cytokine, we hypothesized that butyrate alleviates these colonic changes in IBS models. The visceral pain was assessed by electrophysiologically measuring the threshold of abdominal muscle contractions in response to colonic distention. Colonic permeability was determined by measuring the absorbance of Evans blue in colonic tissue. Colonic instillation of sodium butyrate (SB; 0.37−2.9 mg/kg) for 3 days inhibited LPS (1 mg/kg)-induced visceral allodynia and colonic hyperpermeability dose-dependently. Additionally, the visceral changes induced by repeated WAS (1 h for 3 days) or CRF (50 µg/kg) were also blocked by SB. These effects of SB in the LPS model were eliminated by compound C, an AMPK inhibitor, or GW9662, a PPAR-γ antagonist, N^G^-nitro-L-arginine methyl ester, a NO synthesis inhibitor, naloxone or sulpiride. SB attenuated visceral allodynia and colonic hyperpermeability in animal IBS models. These actions may be AMPK and PPAR-γ dependent and also mediated by the NO, opioid and central dopamine D_2_ pathways. Butyrate may be effective for the treatment of IBS.

## Introduction

Stress-induced altered visceral sensorimotor function is known to be a significant contributor to the pathophysiology of irritable bowel syndrome (IBS)^1^. As the visceral changes by stress are eliminated by a corticotropin-releasing factor (CRF) antagonist, CRF may be a crucial molecule in IBS^2^.

Additionally, it has been recently recognized that the impaired gut barrier associated with abnormal immune response also plays a significant role in IBS^1^. Increased circulatory levels of proinflammatory cytokines and lipopolysaccharide (LPS) are detected in IBS^3,4^, and higher symptom severity is correlated with higher cytokine response induced by LPS in peripheral blood mononuclear cells^4^. We have shown previously that LPS injection induced visceral allodynia and colonic hyperpermeability in rats *via* the peripheral CRF, Toll-like receptor 4 (TLR4), interleukin (IL)-1 and IL-6 pathways^5,6^, which is thought to simulate the pathophysiology of IBS.

At the same time, repeated water avoidance stress (WAS), a well-known animal IBS model, or peripheral injection of CRF also induces these visceral changes *via* similar pathways to LPS^6,7^. In this context, CRF signalling activated by stress (LPS or repeated WAS) possibly induces these changes by modulating TLR4-cytokine signalling, and we considered that it is one of the important mechanisms of IBS^6^. Thus, suppression of the cytokine signalling may be effective for the treatment of this disease.

Butyrate is one of the short-chain fatty acids (SCFAs), which are the main metabolites produced by bacterial fermentation of dietary fiber and is a primary energy source for colonocytes. In addition, it regulates immune function, and exerts the suppressive effects of proinflammatory cytokines^8,9^. In this context, butyrate may be expected to improve visceral changes in these animal IBS models *via* the inhibition of cytokine signalling.

However, the effects of butyrate on visceral functions possibly related to the pathophysiology of IBS have been controversial so far. Rectal enema of butyrate decreases pain in response to rectal balloon distention in healthy human volunteers^10^. Moreover, butyrate reduced colonic paracellular permeability and enhanced the barrier *in vitro*^11,12^. In contrast, several researchers showed that the rectal instillation of butyrate aggravates visceral pain in non-stressed rats^13–16^, and none of the studies has shown that it exerts beneficial effects on visceral function in an animal IBS model.

In this study, we attempted to determine the effects of the colonic instillation of butyrate on visceral sensation and colonic permeability in rat IBS models, i.e. LPS, repeated WAS or CRF, to explore the possibility of therapeutic application of butyrate in IBS.

## Methods

### Animals

Adult male Sprague-Dawley rats (Charles River Laboratory, Atsugi, Japan) weighing about 300 g were used. The animals were group-housed (three to four rats per cage) in a regulated environment with illumination (12 h light/dark cycle) and temperature (23 °C−25 °C). Food (Solid rat chow, Oriental Yeast, Tokyo, Japan) and water were given *ad libitum*.

### Chemicals

Sodium butyrate (SB; Fujifilm Wako Pure Chemical Corporation, Osaka, Japan) was dissolved in phosphate-buffered saline (PBS; 0.14 M NaCl, 2.7 mM KCl, 10 mM Na_2_HPO_4_ and 1.8 mM KH_2_PO_4_). LPS obtained from *Escherichia coli* with serotype 055:B5 (Sigma-Aldrich, St. Louis, MO, USA), a rat/human CRF (Peptide Institute, Inc., Asagi, Japan), N^G^-nitro-L-arginine methyl ester (L-NAME), naloxone hydrochloride and domperidone (Fujifilm Wako Pure Chemical) were dissolved in normal saline. Compound C (dorsomorphin; LC Laboratories, Inc., Woburn, MA, USA), GW9662 (Focus Biomolecules, Plymouth Meeting, PA, USA) and sulpiride (Fujifilm Wako Pure Chemical) were dissolved in dimethyl sulfoxide (Fujifilm Wako Pure Chemical). The doses and routes of administration of the chemicals were determined according to previous publications^5,6,17–20^.

### Measuring visceral sensation

The conscious rats underwent colonic balloon distention to induce abdominal muscle contractions (visceromotor response, VMR), which were measured by an electromyogram (EMG). This method was previously validated as a quantitative measure of visceral nociception^21^. We evaluated the VMR threshold, defined as the volume (ml) of the distended balloon in the current study, and the experiments were performed as described previously^6^. The method of measurement was described briefly in the following.

### Electrodes implantation and colonic distention balloon placement

Under isoflurane anesthesia, a small skin incision was made for the insertion of EMG electrodes (Teflon-coated stainless steel, 0.05 mm diameter) into the left side external oblique muscle in non-fasted rats. The electrodes were fixed to the muscle and the incised skin by cyanoacrylate instant adhesive. Then, the electrode leads were externalized directly through this closed incision without a subcutaneous (s.c.) tunnel and threaded through a urethane tube. Analgesics or antibiotics were not administered. A distention balloon (6-Fr disposable silicon balloon-urethral catheter, JU-SB0601; Terumo Corporation, Tokyo, Japan) was placed intra-anally with the distal end positioned 2 cm proximal to the anus.

### Colonic distention and abdominal muscle contraction measurement

**Figure 1:**
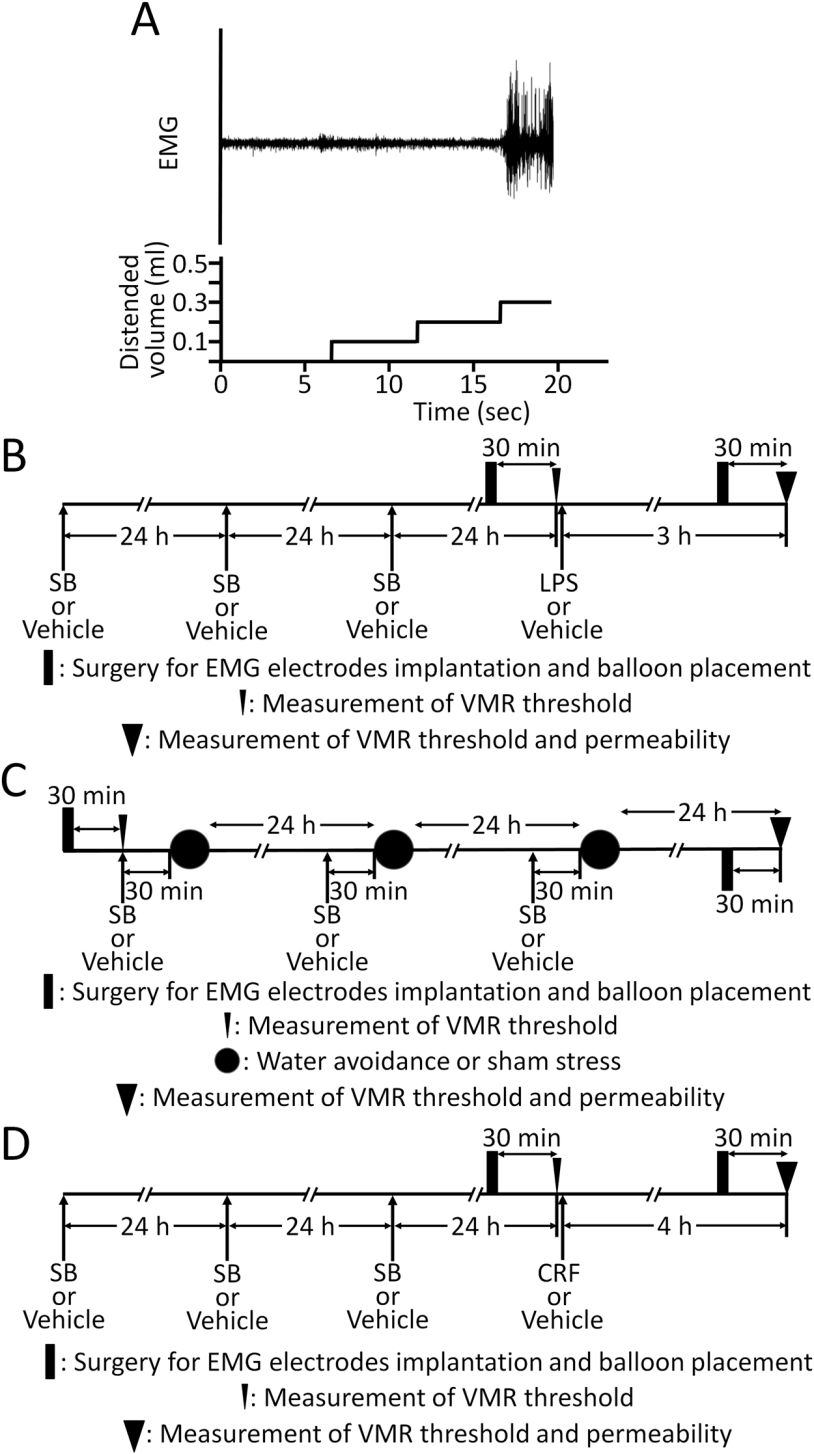
(**A**) Threshold of VMR determined by the distended balloon volume (ml) inducing apparent sustained abdominal muscle contractions. Demonstrable EMG recording is depicted. The threshold of VMR was 0.3 ml in this animal. (**B**) Schematic representation of the experimental protocol to explore the effects of SB on LPS-induced visceral changes. Colonic instillation of SB or the vehicle was performed for 3 consecutive days. The basal VMR threshold was measured 30 min after surgery for implanting EMG electrodes and placing the balloon, i.e. 24 h after the last enema. Then, LPS (1 mg/kg) or the vehicle was administered. Later, surgery and balloon placement were performed again, and the threshold was measured 3 h after injection followed by the measurement of colonic permeability. (**C**) Protocol for determining the effects of SB on repeated WAS-induced visceral changes. Thirty minutes after SB or the vehicle enema, the rats were subjected to either WAS or sham stress for 1 h. These treatments, i.e. enema and stress, were performed for 3 consecutive days. The basal threshold was measured before the initial treatments. The second threshold measurement was performed 24 h after the last stress session followed by the measurement of colonic permeability. (**D**) Effects of SB on CRF (50 µg/kg)-induced visceral changes were also determined. The measurements of the second threshold and colonic permeability were examined 4 h after injection of CRF or the vehicle.

After the electrodes were fixed and the balloon was inserted, the rats were placed in Bollmann cages. The electrode leads were then connected to an EMG amplifier, and the signals were recorded by a PowerLab system (AD Instruments, Colorado Springs, CO, USA). Colonic distention was performed using the ascending method of limits paradigm with phasic distention by inflating the balloon by water using a syringe. The distention was increased progressively in 0.1 ml increments every 5 s until significant abdominal muscle contractions, i.e. VMR, were detected. The VMR threshold was defined as the distended balloon volume (ml) inducing VMR (Fig. 1A). The threshold was assessed twice (2-min interval), and the mean was calculated for each individual animal. The percentage change threshold was calculated as the threshold value after treatment divided by the basal threshold value and multiplied by 100.

### Measurement of colonic permeability

Colonic permeability measurement was performed as described previously^6^. Briefly, the rats were anaesthetised by the administration of the mixture of medetomidine hydrochloride (Orion Pharma Ltd., Dhaka, Bangladesh; 0.15 mg/kg), midazolam (Sandoz, Tokyo, Japan; 2 mg/kg) and butorphanol tartrate (Meiji Seika Pharma, Tokyo, Japan; 2.5 mg/kg) intraperitoneally (i.p.) and underwent laparotomy. The colon was ligated at the junction with the cecum, and an open-tipped catheter (3-Fr, Atom, Tokyo, Japan) was inserted into the proximal colon through the hole made by a puncture using needle. The colon was gently flushed with PBS using the catheter in order to wash out all stools, and later, another ligation was added on the colon at approximately 4 cm from the proximal one. Then, 1 ml of 1.5% Evans blue in PBS was instilled into the colon segment through the catheter. After 15 min, the rats were euthanized by terminal exsanguination under deep isoflurane anesthesia. The colons were excised, washed with PBS and 6 mM N-acetyl-cysteine, and placed in 2 ml N,N-dimethylformamide for 12 h. Permeability was calculated by measuring the Evans blue concentration in the supernatant using a spectrophotometer at 610 nm.

### Butyrate enema

The non-anesthetized rats placed in Bollmann cages underwent intra-anal insertion of a catheter (JU-SB0601; Terumo) with the distal end positioned 7 cm proximal to the anus. As the catheter was customized, the solution can diffuse from the distal end of the catheter. The rats received 0.5 ml SB solution (2, 6 and 16 mmol/l) once daily, i.e. at doses of 0.37, 1.1 and 2.9 mg/kg/day, for 3 consecutive days through the catheter. The controls were treated with the vehicle (PBS).

### Stress protocol

Exposure to WAS was performed as described previously^22^. Briefly, rats were individually placed on a plastic platform (height, 8 cm; length, 6 cm; width, 6 cm) positioned in the middle of a plastic cage, which was filled with water up to 7 cm of the platform height. Control animals were individually placed in the same plastic cage, which was not filled with water (sham stress).

### Experimental procedures

Six groups of five to eleven rats were used. After 24 h from the last colonic instillation of SB with different concentrations or PBS, the basal VMR threshold was assessed. Next, the electrodes and distention balloon were removed, and either the vehicle or LPS (1 mg/kg) was s.c. injected (Fig. 1B). The rats were returned to their home cages, and after 3 h, the second measurement of threshold was implemented followed by the measurement of colonic permeability^5^.

Next, four groups of five to six rats were used to evaluate the effects of SB on WAS model (Fig. 1C). First, the basal threshold was measured. Then SB or the vehicle enema followed by WAS or sham stress for 1 h was implemented for 3 consecutive days. The measurement of the second threshold followed by colonic permeability was performed 24 h after undergoing the last stress session^7^.

The effects of SB on CRF model were also tested using four groups of five to six rats. The second threshold was measured 4 h after injection of CRF (50 µg/kg, i.p.) or the vehicle followed by the measurement of colonic permeability (Fig. 1D)^6^.

Next, to evaluate the mechanisms of actions of SB, the effects of compound C (2 mg/kg s.c.), GW9662 (3 mg/kg s.c.), L-NAME (10 mg/kg i.p.), naloxone (1 mg/kg s.c.), sulpiride (200 mg/kg s.c.) or domperidone (10 mg/kg s.c.) were tested. The groups in these experiments consisted of five to six rats. These drugs were given together with SB or the vehicle enema.

### Statistical analysis

Statistical analyses were performed using SYSTAT 13 software (Systat Software, Chicago, IL, USA). Data were presented as means ± SEM. Multiple comparisons were performed by one- or two-way analysis of variance (ANOVA) followed by Tukey’s honestly significant difference (HSD) test. Comparisons between the two groups were performed using Student’s *t*-test.

### Ethical statement

For all studies, approval was obtained from the Research and Development and Animal Care Committees at Asahikawa Medical University (#17149, approved on August 2, 2017).

### Ethical approval

All applicable international, national, and/or institutional guidelines for the care and use of animals were followed.

## Results

### SB blocked LPS-induced visceral allodynia and colonic hyperpermeability

**Figure 2:**
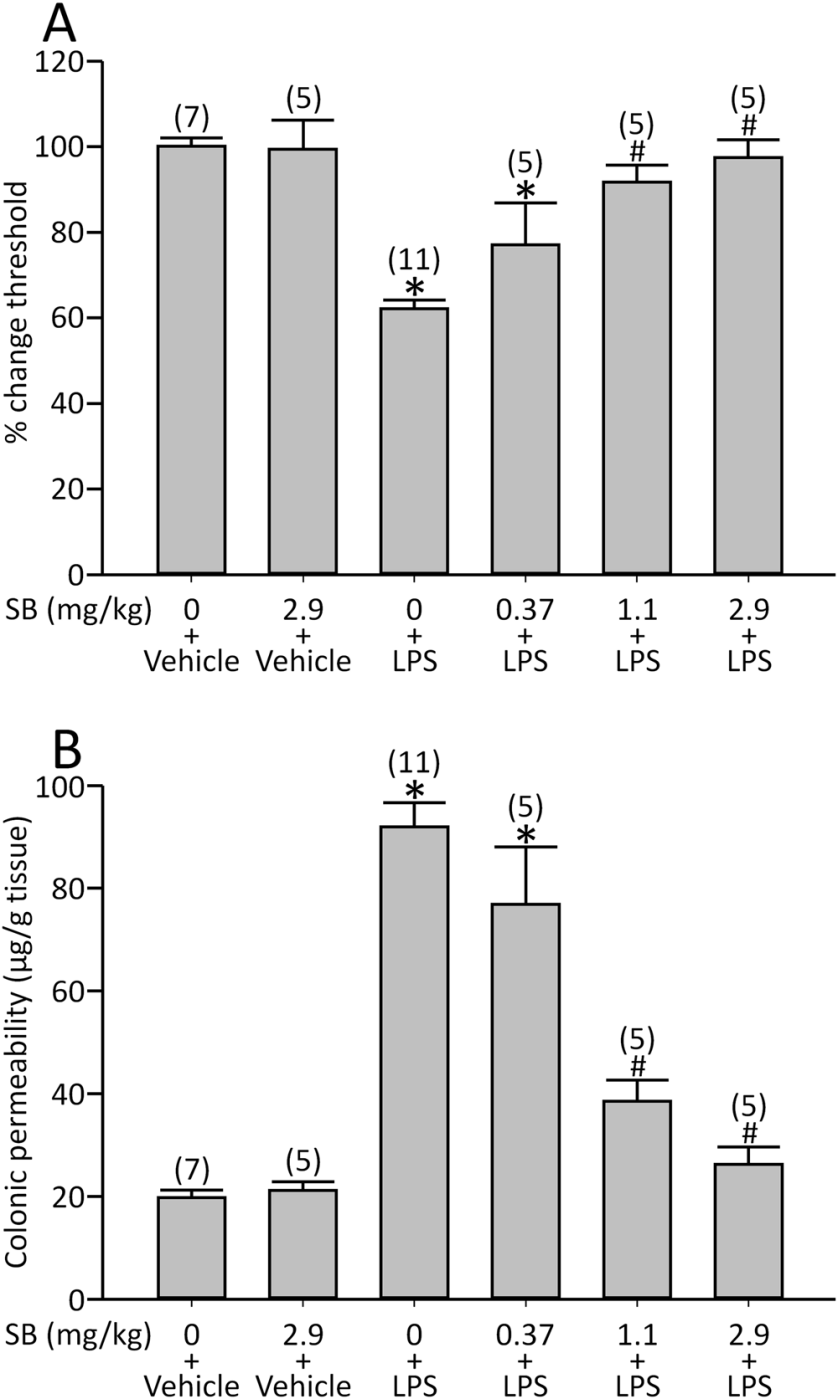
Effects of SB on LPS-induced visceral allodynia and colonic hyperpermeability. (**A**) LPS significantly reduced the threshold of VMR, and SB dose-dependently reversed this response. (**B**) LPS increased colonic permeability, which was also blocked by SB. *p < 0.05 vs. vehicle (SB 0) + vehicle, ^#^p < 0.05 vs. vehicle (SB 0) + LPS by one-way ANOVA followed by Tukey’s HSD test. Each column represents mean ± SEM. The number of rats examined is shown in parentheses (*n* = 5−11).

SB inhibited LPS-induced visceral allodynia in a dose-responsive manner [F(5,32) = 15.1, *p* < 0.05; Fig. 2A]. SB (2.9 mg/kg) fully reversed the threshold change by LPS. This dose of SB *per se* did not change the basal threshold of VMR (ml), i.e. before injection of LPS or the vehicle (0.62 ± 0.026 for SB, *n* = 10 vs. 0.63 ± 0.017 for vehicle, *n* = 18, *p* > 0.05).

Additionally, SB inhibited LPS-induced colonic hyperpermeability dose-dependently [F(5,32) = 42.2, *p* < 0.05; Fig. 2B]. According to these results, we employed 2.9 mg/kg SB for the following experiments.

### SB eliminated repeated WAS- or CRF-induced visceral allodynia and colonic hyperpermeability

**Figure 3:**
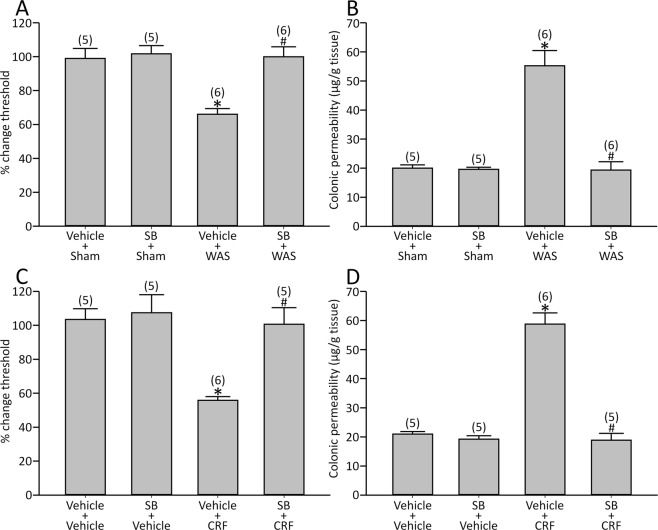
Effect of SB on repeated WAS- or CRF-induced visceral changes. WAS induced visceral allodynia and colonic hyperpermeability, which were reversed by SB (**A**,**B**). SB also blocked CRF-induced visceral changes (**C**,**D**). Sham, sham stress. *p < 0.05 vs. vehicle + sham or vehicle + vehicle, ^#^p < 0.05 vs. vehicle + WAS or vehicle + CRF by two-way ANOVA followed by Tukey’s HSD test. Each column represents mean ± SEM. The number of rats examined is shown in parentheses (*n* = 5−6).

Visceral changes induced by repeated WAS were abolished by SB [visceral sensation: effect of WAS: F(1,18) = 11.6, *p* < 0.05, effect of SB: F(1,18) = 12.8, *p* < 0.05 and interaction between WAS and SB: F(1,18) = 9.38, *p* < 0.05; colonic permeability: effect of WAS: F(1, 18) = 27.2, *p* < 0.05, effect of SB: F(1,18) = 29.2, *p* < 0.05 and interaction between WAS and SB: F(1,18) = 27.9, *p* < 0.05; Fig. 3A,B].

SB also blocked these CRF-induced visceral changes [visceral sensation: effect of CRF: F(1,17) = 12.9, *p* < 0.05, effect of SB: F(1,17) = 10.4, *p* < 0.05 and interaction between CRF and SB: F(1,17) = 7.29, *p* < 0.05; colonic permeability: effect of CRF: F(1,17) = 52.2, *p* < 0.05, effect of SB: F(1,17) = 64.6, *p* < 0.05 and interaction between CRF and SB: F(1,17) = 54.2, *p* < 0.05; Fig. 3C,D].

### Compound C reversed the effects of SB on the LPS model

As SCFAs were reported to activate AMP-activated protein kinase (AMPK) signalling^23^, the role of AMPK on the effects of SB was determined. First, we evaluated the effects of compound C, an AMPK inhibitor, on the basal threshold, and the LPS-induced visceral allodynia and colonic hyperpermeability. Three s.c. injections of compound C altered neither the basal threshold (0.60 ± 0.020 ml for compound C, *n* = 10 vs. 0.60 ± 0.015 ml for vehicle, *n* = 10, *p* > 0.05) nor the visceral response induced by LPS [visceral sensation: effect of compound C: F(1,16) = 0.028, *p* > 0.05, effect of LPS: F(1,16) = 122.8, *p* < 0.05 and interaction between compound C and LPS: F(1,16) = 0.46, *p* > 0.05; colonic permeability: effect of compound C: F(1,16) = 0.017, *p* > 0.05, effect of LPS: F(1,16) = 37.9, *p* < 0.05 and interaction between compound C and LPS: F(1,16) = 0.025, *p* > 0.05].

**Figure 4:**
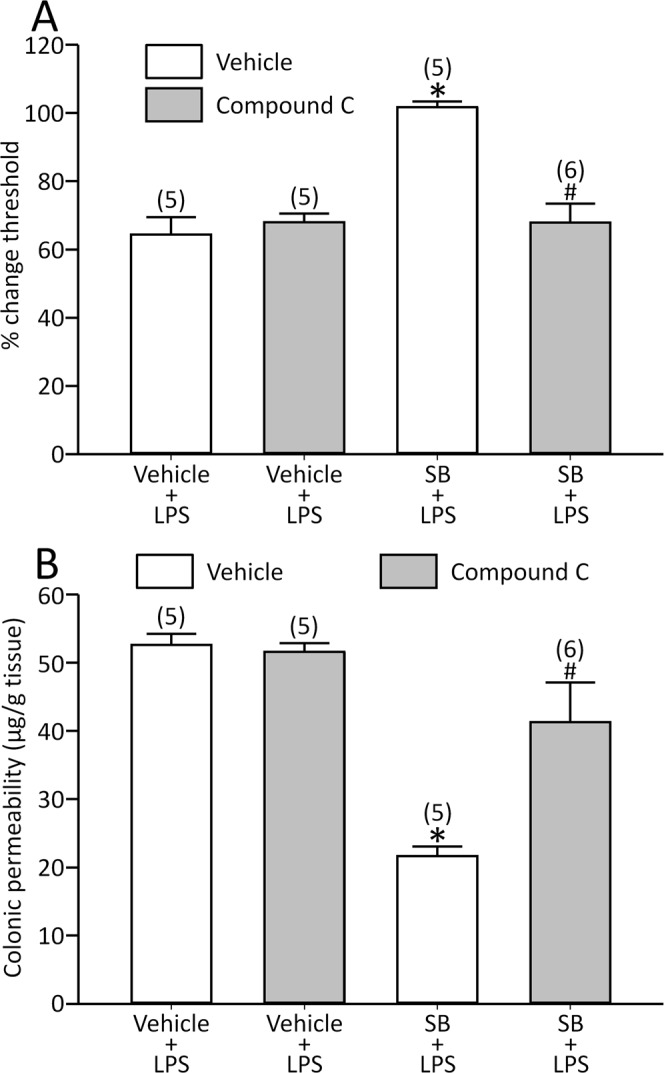
Compound C reversed the effects of SB on LPS-induced visceral allodynia (**A**) and colonic hyperpermeability (**B**). *p < 0.05 vs. vehicle + vehicle + LPS, ^#^p < 0.05 vs. vehicle + SB + LPS by two-way ANOVA followed by Tukey’s HSD test. Each column represents mean ± SEM. The number of rats examined is shown in parentheses (*n* = 5−6).

Then we performed separate series of experiments to explore the role of AMPK signalling on the effects of SB, and compound C reversed the effects of SB on the LPS model [visceral sensation: effect of compound C: F(1,17) = 12.3, *p* < 0.05, effect of SB: F(1,17) = 18.8, *p* < 0.05 and interaction between compound C and SB: F(1,17) = 19.0, *p* < 0.05; colonic permeability: effect of compound C: F(1,17) = 6.74, *p* < 0.05, effect of SB: F(1,17) = 33.0, *p* < 0.05 and interaction between compound C and SB: F(1,17) = 8.27, *p* < 0.05; Fig. 4A,B].

### GW9662 eliminated the effects of SB on the LPS model

A butyrate-releasing derivative was shown to activate peroxisome proliferator-activated receptor-γ (PPAR-γ)^24^, and its role was explored. GW9662, a PPAR-γ antagonist, modified neither the basal threshold (0.60 ± 0.016 ml for GW9662, *n* = 12 vs. 0.60 ± 0.025 ml for vehicle, *n* = 10, *p* > 0.05) nor the response induced by LPS [visceral sensation: effect of GW9662: F(1,18) = 0.14, *p* > 0.05, effect of LPS: F(1,18) = 58.3, *p* < 0.05 and interaction between GW9662 and LPS: F(1,18) = 0.015, *p* > 0.05; colonic permeability: effect of GW9662: F(1,18) = 0.044, *p* > 0.05, effect of LPS: F(1,18) = 169.5, *p* < 0.05 and interaction between GW9662 and LPS: F(1,18) = 0.12, *p* > 0.05].

**Figure 5:**
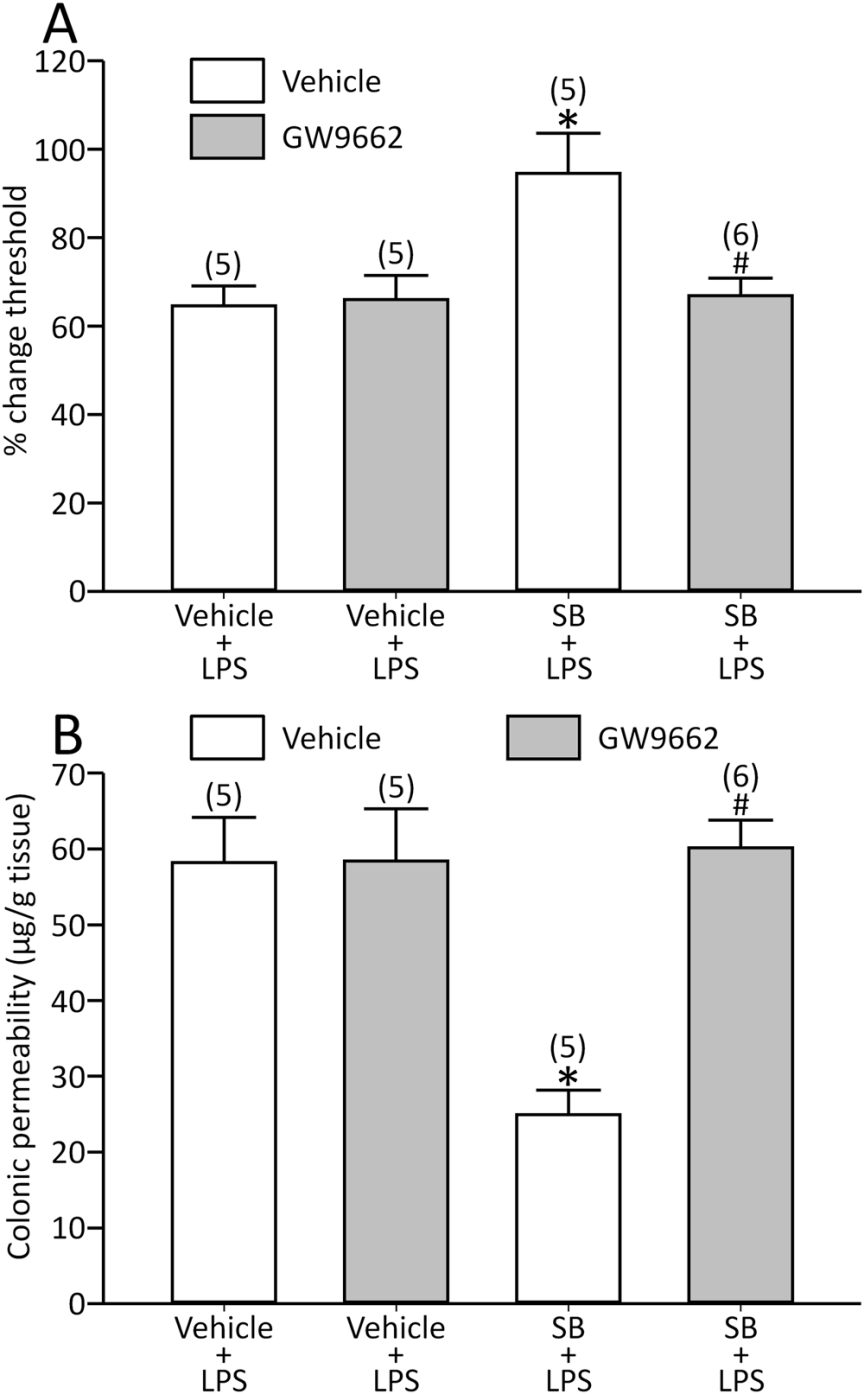
GW9662 blocked the effects of SB on LPS-induced visceral allodynia (**A**) and colonic hyperpermeability (**B**). *p < 0.05 vs. vehicle + vehicle + LPS, ^#^p < 0.05 vs. vehicle + SB + LPS by two-way ANOVA followed by Tukey’s HSD test. Each column represents mean ± SEM. The number of rats examined is shown in parentheses (*n* = 5−6).

The drug abolished the effects of SB [visceral sensation: effect of GW9662: F(1,17) = 4.95, *p* < 0.05, effect of SB: F(1,17) = 6.82, *p* < 0.05 and interaction between GW9662 and SB: F(1,17) = 6.05, *p* < 0.05; colonic permeability: effect of GW9662: F(1,17) = 12.3, *p* < 0.05, effect of SB: F(1,17) = 9.78, *p* < 0.05 and interaction between GW9662 and SB: F(1,17) = 12.0, *p* < 0.05; Fig. 5A,B].

### L-NAME or naloxone reversed the effects of SB on the LPS model

Butyrate was shown to increase nitric oxide (NO) production from macrophages^25^, and NO is known to modulate pain response^26,27^. Therefore, we determined the role of NO. L-NAME, an NO synthesis inhibitor, did not alter either the basal threshold (0.60 ± 0.017 ml for L-NAME, *n* = 10 vs. 0.61 ± 0.016 ml for vehicle, *n* = 10, *p* > 0.05) or the response by LPS [visceral sensation: effect of L-NAME: F(1,16) = 0.24, *p* > 0.05, effect of LPS: F(1,16) = 88.7, *p* < 0.05 and interaction between L-NAME and LPS: F(1,16) = 0.152, *p* > 0.05; colonic permeability: effect of L-NAME: F(1,16) = 0.027, *p* > 0.05, effect of LPS: F(1,16) = 84.7, *p* < 0.05 and interaction between L-NAME and LPS: F(1,16) = 0.0001, *p* > 0.05].

**Figure 6:**
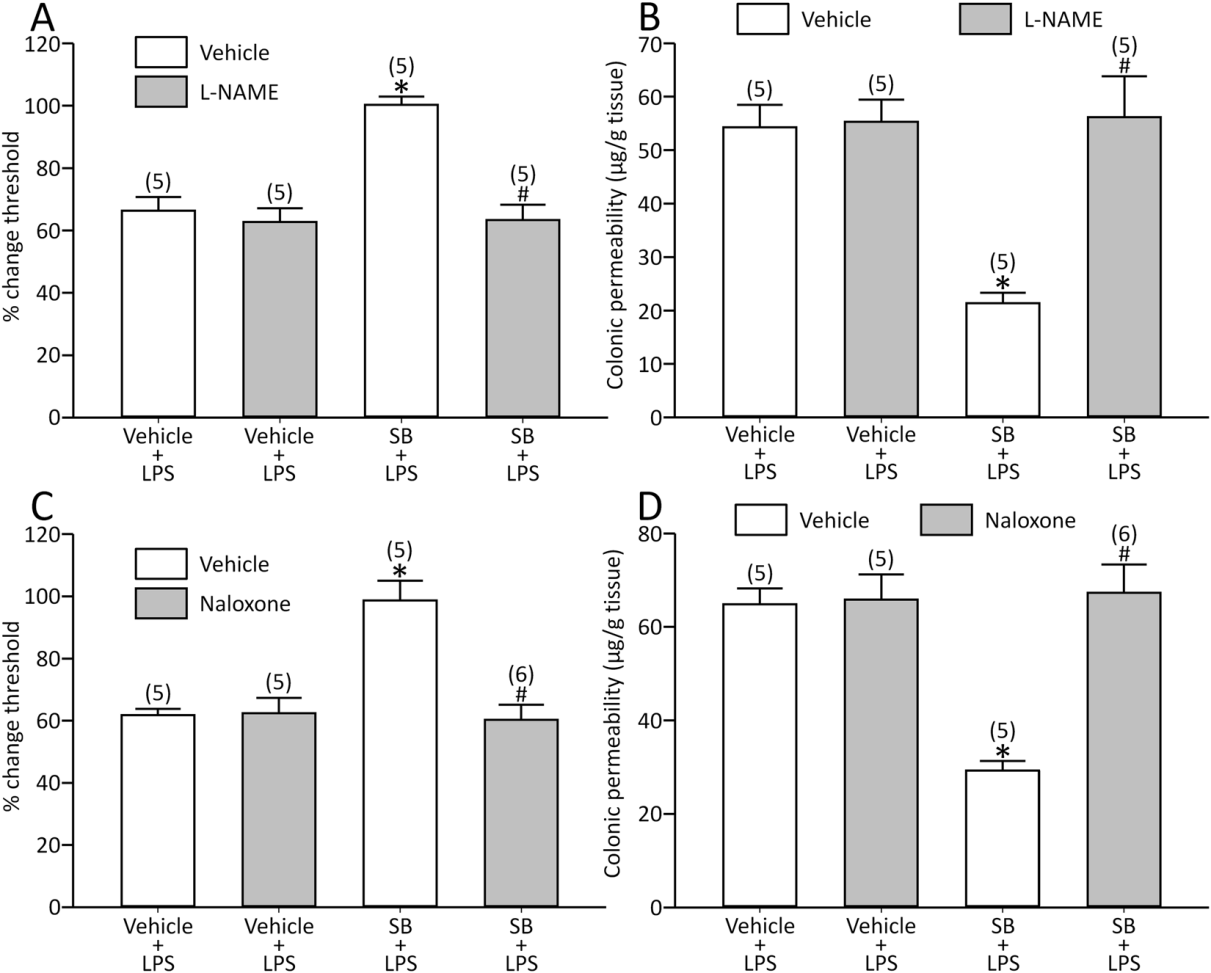
L-NAME abolished the effects of SB on LPS-induced visceral changes (**A**,**B**). Naloxone also reversed the effects of SB (**C**,**D**). *p < 0.05 vs. vehicle + vehicle + LPS, ^#^p < 0.05 vs. vehicle + SB + LPS by two-way ANOVA followed by Tukey’s HSD test. Each column represents mean ± SEM. The number of rats examined is shown in parentheses (*n* = 5−6).

At the same time, the drug fully reversed the effects of SB [visceral sensation: effect of L-NAME: F(1,16) = 22.9, *p* < 0.05, effect of SB: F(1,16) = 16.7, *p* < 0.05 and interaction between L-NAME and SB: F(1,16) = 15.5, *p* < 0.05; colonic permeability: effect of L-NAME: F(1,16) = 13.2, *p* < 0.05, effect of SB: F(1,16) = 10.6, *p* < 0.05 and interaction between L-NAME and SB: F(1,16) = 11.7, *p* < 0.05; Fig. 6A,B].

It is well known that opioid signalling is involved in visceral sensation^28^, and its role on the effects of butyrate was determined. Naloxone, an opioid receptor antagonist, altered neither the basal threshold (0.62 ± 0.013 ml for naloxone, *n* = 10 vs. 0.61 ± 0.018 ml for vehicle, *n* = 10, *p* > 0.05) nor the response by LPS [visceral sensation: effect of naloxone: F(1,16) = 0.059, *p* > 0.05, effect of LPS: F(1,16) = 99.6, *p* < 0.05 and interaction between naloxone and LPS: F(1,16) = 0.033, *p* > 0.05; colonic permeability: effect of naloxone: F(1,16) = 0.024, *p* > 0.05, effect of LPS: F(1,16) = 189.8, *p* < 0.05 and interaction between naloxone and LPS: F(1,16) = 0.03, *p* > 0.05].

Additionally, naloxone abolished the effects of SB [visceral sensation: effect of naloxone: F(1,17) = 15.2, *p* < 0.05, effect of SB: F(1,17) = 12.9, *p* < 0.05 and interaction between naloxone and SB: F(1,17) = 16.3, *p* < 0.05; colonic permeability: effect of naloxone: F(1,17) = 17.0, *p* < 0.05, effect of SB: F(1,17) = 13.0, *p* < 0.05 and interaction between naloxone and SB: F(1,17) = 15.3, *p* < 0.05; Fig. 6C,D].

### Sulpiride abolished but domperidone did not alter the effects of SB on the LPS model

We have previously demonstrated that central dopamine signalling is an important modulator of visceral pain^29^, and its role on the effects of butyrate was determined. Sulpiride, a dopamine D_2_ receptor antagonist, did not modify the basal threshold (0.62 ± 0.015 ml for sulpiride, *n* = 10 vs. 0.62 ± 0.019 ml for vehicle, *n* = 10, *p* > 0.05) and the response by LPS [visceral sensation: effect of sulpiride: F(1,16) = 0.123, *p* > 0.05, effect of LPS: F(1,16) = 77.7, *p* < 0.05 and interaction between sulpiride and LPS: F(1,16) = 0.246, *p* > 0.05; colonic permeability: effect of sulpiride: F(1,16) = 1.83, *p* > 0.05, effect of LPS: F(1,16) = 190.9, *p* < 0.05 and interaction between sulpiride and LPS: F(1,16) = 1.93, *p* > 0.05].

**Figure 7:**
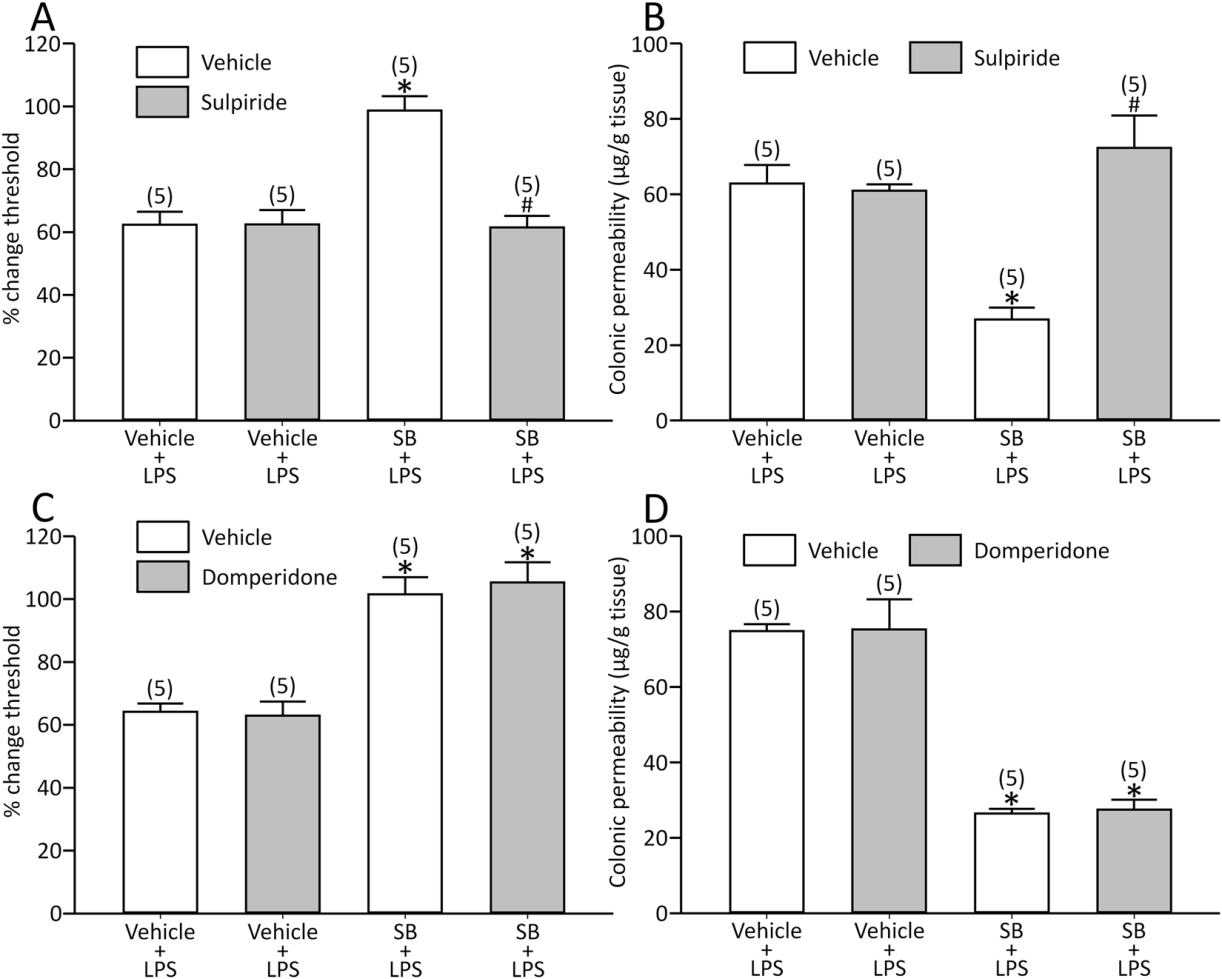
Sulpiride eliminated the effects of SB on LPS-induced visceral changes (**A** and **B**), whereas domperidone did not modify the effects (**C** and **D**). *p < 0.05 vs. vehicle + vehicle + LPS, ^#^p < 0.05 vs. vehicle + SB + LPS by two-way ANOVA followed by Tukey’s HSD test. Each column represents mean ± SEM. The number of rats examined is shown in parentheses (*n* = 5).

The drug reversed the effects of SB [visceral sensation: effect of sulpiride: F(1,16) = 18.8, *p* < 0.05, effect of SB: F(1,16) = 17.2, *p* < 0.05 and interaction between sulpiride and SB: F(1,16) = 19.0, *p* < 0.05; colonic permeability: effect of sulpiride: F(1,16) = 17.2, *p* < 0.05, effect of SB: F(1,16) = 5.52, *p* < 0.05 and interaction between sulpiride and SB: F(1,16) = 20.5, *p* < 0.05; Fig. 7A,B].

Domperidone, a peripheral dopamine D_2_ receptor antagonist, did not modify the basal threshold (0.64 ± 0.026 ml for domperidone, *n* = 10 vs. 0.63 ± 0.013 ml for vehicle, *n* = 10, *p* > 0.05) and the response by LPS [visceral sensation: effect of domperidone: F(1,16) = 0.256, *p* > 0.05, effect of LPS: F(1,16) = 108.8, *p* < 0.05 and interaction between domperidone and LPS: F(1,16) = 0.047, *p* > 0.05; colonic permeability: effect of domperidone: F(1,16) = 0.228, *p* > 0.05, effect of LPS: F(1,16) = 111.8, *p* < 0.05 and interaction between domperidone and LPS: F(1,16) = 0.047, *p* > 0.05].

At the same time, the drug did not modify the effects of SB [visceral sensation: effect of domperidone: F(1,16) = 0.067, *p* > 0.05, effect of SB: F(1,16) = 64.8, *p* < 0.05 and interaction between domperidone and SB: F(1,16) = 0.26, *p* > 0.05; colonic permeability: effect of domperidone: F(1,16) = 0.03, *p* > 0.05, effect of SB: F(1,16) = 124.8, *p* < 0.05 and interaction between domperidone and SB: F(1,16) = 0.005, *p* > 0.05; Fig. 7C,D].

## Discussion

The effects of butyrate on visceral sensation are controversial. Bourdu *et al*.^13^ reported that SB enema induced visceral hypersensitivity in rats for the first time and advocated that this response by SB could be used for an experimental animal model of IBS. After that, this phenomenon has been well reconfirmed by several other researchers, and it is now recognized as one of the common rat IBS models^14–16^. In contrast, a few human studies showed that butyrate improved abdominal pain in patients with IBS^30,31^. Additionally, repeated WAS decreases butyrate-producing microbiota, and visceral hypersensitivity induced by stress is alleviated by the supplementation of butyrate-producing bacteria in rats^32^, which may be indirect evidence suggesting the ameliorative effect of butyrate in visceral pain.

In the study of Bourdu *et al*.^13^, the rats underwent 1 ml SB enema twice daily for 3 consecutive days, and the tested concentrations of SB solution ranged from 8 to 1000 mmol/l. Under this protocol, SB induced visceral allodynia in a dose-responsive manner. Meanwhile, butyrate concentrations in the cecal fluid and colonic contents of rats, pigs and monkeys were reported to be 3 to 7 mmol/l when diets contained little or no fermentable dietary fiber, and as high as 40 mmol/l when the diets provided ample fermentable fiber^33^. Given the evidence above, the concentrations of SB solution in that study seemed to be extremely high. Thus, we used the physiologic concentration of SB solution for enema, i.e. 2, 6 and 16 mmol/l. The doses of SB using these solutions were 0.37, 1.1 and 2.9 mg/kg/day, whereas the doses in that study ranged from 8.4 to 1048 mg/kg/day, which means that the highest tested dose in the current study equaled only about one third of the minimum tested dose by Bourdu *et al*.

The current study showed for the first time that SB abolished visceral allodynia in rat IBS models, which was completely different from the findings by the previous studies above. Our results strongly suggest that a high dose of SB may induce visceral hypersensitivity, and physiologic concentrations of SB may improve visceral pain. Intraluminal administration of physiologic doses of butyrate into the distal colon for 7 days decreases visceral pain and discomfort in response to colonic distention in healthy humans^10,34^, which may further support our results and the notion above.

At the same time, we also found that SB improved colonic barrier. Previous studies showed that butyrate in physiologic concentrations can enhance intestinal barrier function, but high-dose butyrate disrupts the barrier using Caco-2 cells *in vitro*^35^. These findings strongly suggest that an adequate dose of butyrate may improve visceral function.

We have recently demonstrated that LPS-, repeated WAS- or CRF-induced visceral allodynia and colonic hyperpermeability were mediated *via* peripheral CRF, TLR4 and the proinflammatory cytokine system^5–7^. The speculated mechanisms of the visceral changes in these IBS models are considered as follows^6^. Activating peripheral CRF receptors by stress stimulates TLR4 to alter tight junction (TJ) proteins^36^, thereby inducing colonic hyperpermeability. Impaired gut barrier induces bacterial translocation leading to increased LPS to trigger to release proinflammatory cytokines by activating TLR4, which induces visceral allodynia possibly through the activation of visceral afferent neurons^37^. At the same time, the cytokine also increases gut permeability *via* modifying TJ proteins^38^. Additionally, LPS not only stimulates TLR4 but also activates peripheral CRF receptors^5^. Thus, peripheral CRF and TLR4-cytokine signalling may develop a vicious cycle activating each other to induce these visceral changes.

In this scenario, visceral allodynia is considered to result from colonic hyperpermeability. In the current and our previous studies showed that visceral allodynia occurred associated with colonic hyperpermeability with no exception^6,17,18,39^. Moreover, Creekmore *et al*.^40^, demonstrated a positive correlation between the magnitude of visceral pain and paracellular permeability in repeated WAS model, and knockdown of occludin, one of the TJ proteins, induced intestinal hyperpermeability with visceral hypersensitivity. These results further support the notion above. Thus, SB may inhibit peripheral CRF-TLR4-cytokine signaling to improve colonic barrier followed by inhibition of visceral allodynia.

Actually, butyrate was reported to inhibit the expression of proinflammatory cytokines triggered by interferon-γ in RAW 264.7 cells^41^. Moreover, it was also shown that high-fat diet impaired gut barrier with increased serum level of LPS and upregulated the TLR4 gene and proinflammatory cytokines in the liver, which were improved by the intragastric administration of SB in mice^42^.

SCFAs are known to activate AMPK in the liver and muscles^23^. In addition, we have very recently shown that metformin, an AMPK activator, blocked the visceral changes in the same IBS models^18^. Thus, we hypothesized that the effects of butyrate are mediated *via* AMPK, and it actually happened, i.e. compound C reversed the effects of SB. Several *in vitro* studies proved that LPS-induced inflammatory response was inhibited by AMPK^43,44^. In this context, the effects of SB may result from the suppression of cytokine signalling *via* AMPK.

At the same time, it was reported that butyramide, a butyrate-releasing derivative exerts an anti-inflammatory effect *via* the upregulation of PPAR-γ in dextran sulphate sodium-induced murine colitis^24^. PPAR-γ inhibits the expression of various cytokines in macrophages^45^, and we have also confirmed previously that activating PPAR-γ by pioglitazone abolished the visceral changes in these IBS models^17^. Moreover, butyrate reduces colonic paracellular permeability by PPAR-γ activation in HT-29 cells^11^. In the current study, the effects of SB were reversed by PPAR-γ antagonist, suggesting that butyrate may inhibit cytokine signalling *via* PPAR-γ to exert the effects.

It is known that there exists a link between AMPK and PPAR-γ. It was shown that LPS increased the expression of TLR4 *via* the suppression of PPAR-γ in endothelial EA.hy926 cells, and the activation of AMPK prevented the increase of TLR4 protein *via* the rescue of the decreased PPAR-γ protein^46^, suggesting that PPAR-γ might be a downstream effector of AMPK. In contrast, several studies showed that PPAR-γ mediates the activation of AMPK^47^. In this context, both signalling can modulate each other. Although both signalling modulated the effects of SB in the current study, we did not determine which signalling was upstream. Further studies are needed to explore this issue.

We also showed that the effects of SB were reversed by L-NAME or sulpiride, but not by domperidone, suggesting that they were mediated *via* NO and central dopamine D_2_ signalling. These findings may support the result that the effects of SB were mediated *via* AMPK signalling, because the antinociceptive effect by metformin in the LPS model was mediated *via* NO and central dopamine D_2_ pathways, which were shown in our previous study^18^.

It has been demonstrated that NO pathway may be both pro-nociceptive and anti-nociceptive^48^. Based on the present study, we would raise a hypothesis that butyrate increases NO production from macrophages^25^, and NO inhibits proinflammatory cytokine genes in various immune cells^49^, thereby improving the IBS model.

Garrido-Gil *et al*.^50^ showed that central dopaminergic depletion increased the level of IL-1β in the colon, suggesting that brain dopamine reduces the vulnerability of gut inflammation. Additionally, butyrate possibly crosses the blood brain barrier^51^, and SB at a dose of 300 mg/kg i.p. protects dopamine neurons to improve the motor deficit in Parkinson’s disease model^52^, suggesting the possibility that SB act centrally to modulate dopamine signalling to exert the effects by suppressing cytokine.

However, we did not think that SB directly act on the brain in the current study for the following reasons. Butyrate is the main energy source for colonocytes^23^, and the majority of the luminal butyrate is consumed in the gut resulting in relatively low concentration of butyrate in portal vein. Then it is metabolized in liver and its concentration becomes lower in systemic circulation^53^. Moreover, the brain uptake of intravenous administration of butyrate was reported to be only less than 0.006% in baboons^54^. Therefore, the amount of brain uptake is considered to be negligible in the current experimental settings using physiologic concentration of SB enema. Butyrate is known to activate vagal afferents^55^, and activation of upper gut-innervating vagal afferents induces the release of dopamine from nigral neurons^56^. In this context, it is reasonable to think that SB may act peripherally and indirectly activate brain dopamine D_2_ receptor.

As it is known that opioid signalling is involved in altered visceral sensory function by stress^28^, the role of opioid receptor was determined. Opioid receptors are expressed in immune cells and modulate cytokine response^57^. We found that naloxone blocked the effects of SB. There is no direct report indicating that butyrate activates opioid signalling, but Pol *et al*.^58^ demonstrated that NO upregulated the µ-opioid receptor gene transcription in mice gut during intestinal inflammation. Moreover, NO stimulated the neuronal release of endogenous opioids to stimulate opioid receptors in the brain and the spinal cord^26,27^. These results suggest that butyrate may activate opioid receptors *via* NO to exert the effects. In this context, both peripheral and central opioid signalling may contribute to the effects of butyrate.

This study has several limitations. We did not show the direct evidence that SB inhibits the production of cytokines. Although the visceral changes observed in these IBS models were mediated *via* IL-1 or IL-6^5–7^, the colonic mucosal levels of the cytokines were not elevated in the current experimental settings (data not shown). Therefore, we could not test the direct effect of SB on cytokine signalling. Since cytokines may act locally to visceral afferents or TJ inducing visceral changes, increased cytokines in the colonic mucosa were not prerequisite for these changes. In other words, activating local cytokine signaling and the elevated level of cytokines are different issues. Moreover, the sources of the cytokines responsible for the visceral changes were not determined. It is known that various cells other than macrophages or monocytes, such as fibroblasts, endothelial cells, neuronal cells and smooth muscle cells, secrete the cytokines^59^. Additionally, the role of opioid receptor subtypes on the effects of butyrate was not determined either. Before determining the precise mechanisms of actions by SB in a molecular or cellular level, we should clear the issue above.

In spite of these limitations, our results clearly showed that butyrate improved visceral changes in IBS models. Recently, several studies have been demonstrated that perturbations of the intestinal microbiota play a role in the pathophysiology of IBS. Although it is not definitely known that an altered microbiota is a cause or a consequence, it may be involved with the changes in intestinal motility, visceral sensation, mucosal barrier and the expression of pattern recognition receptors^60^. The microbiota generates and releases molecules that can signal to distant organs, which may induce these changes. Together with the evidence above, our results suggest that butyrate is one of the signalling molecules between the microbiota and host in the pathophysiology of IBS.

In conclusion, SB enema blocked visceral allodynia and colonic hyperpermeability in rat IBS models, which may be AMPK and PPAR-γ dependent, and mediated by the NO, central dopamine D_2_ and opioid pathways. Butyrate may be useful for the treatment of IBS.

## Data availability

All data generated or analysed during this study this study are included in this published article.
